# Aggressive Mating Behavior in Roosters (*Gallus gallus domesticus*): A Narrative Review of Behavioral Patterns

**DOI:** 10.3390/life15081232

**Published:** 2025-08-03

**Authors:** Mihnea Lupu, Dana Tăpăloagă, Elena Mitrănescu, Raluca Ioana Rizac, George Laurențiu Nicolae, Manuella Militaru

**Affiliations:** Faculty of Veterinary Medicine, University of Agronomic Sciences and Veterinary Medicine of Bucharest, 105 Splaiul Independentei, District 5, 050097 Bucharest, Romania; mihnea.l96@gmail.com (M.L.); elena.mitranescu@fmvb.usamv.ro (E.M.); raluca.rizac@fmvb.usamv.ro (R.I.R.); george-laurentiu.nicolae@fmvb.usamv.ro (G.L.N.); manuella.militaru@fmvb.usamv.ro (M.M.)

**Keywords:** rooster sexual aggression, rooster courtship behavior, breeding hens, traumatic injuries in hens

## Abstract

This review explores sexual aggression in broiler breeder males, aiming to synthesize existing scientific evidence regarding its causes, behavioral manifestations, and consequences, while addressing the genetic, neuroendocrine, and environmental mechanisms involved. Through an extensive analysis of scientific literature, the paper highlights that intensive genetic selection aimed at enhancing growth and productivity has resulted in unintended behavioral dysfunctions. These include the reduction or absence of courtship behavior, the occurrence of forced copulations, and a notable increase in injury rates among hens. Reproductive challenges observed in meat-type breeder flocks, in contrast to those in layer lines, appear to stem from selection practices that have overlooked traits related to mating behavior. Environmental and managerial conditions, including photoperiod manipulation, stocking density, nutritional imbalances, and the use of mixed-sex rearing systems, are also identified as contributing factors to the expression of sexual aggression. Furthermore, recent genetic findings indicate a potential link between inherited neurobehavioral factors and aggressive behavior, with the *SORCS2* gene emerging as a relevant candidate. Based on these insights, the review emphasizes the importance of considering behavioral parameters in breeding programs in order to reconcile productivity objectives with animal welfare standards. Future research may benefit from a more integrative approach that combines behavioral, physiological, and genomic data to better understand and address the multifactorial nature of sexual aggression in poultry systems.

## 1. Introduction

Aggressive behavior of roosters toward hens are rare in natural environments, where the two sexes form separate social hierarchies and male dominance is expressed in a passive manner [1,2,3]. However, it has been observed that males from broiler parental lines exhibit a high frequency of aggression toward females, especially during the display of sexual behavior [4,5,6,7,8,9,10,11,12,13]. This pattern of mating-related aggression has also been observed in slow-growing broiler breeder populations, indicating that the expression of sexual aggressiveness is not limited to fast-growing lines [14]. From a behavioral perspective, males have been described as exhibiting violent sexual behavior, pecking at or chasing hens, which leads to forced copulations [4,6,8,10,11,13,14]. Males show deficiencies in mating behavior, particularly in courtship [4,5,8,11,14,15], that appear to be associated with selection for production traits [16,17,18]. When discussing rooster lines that have been genetically selected for fighting ability in the ring, these males did not exhibit aggressive behavior toward females, suggesting that sexual aggression was not associated with general aggression [7].

Between 1976 and 1977, a study was conducted on the causes of mortality in three flocks of breeder hens belonging to broiler parental lines during the laying phase. Although the term “sexual aggressiveness” was not used at that time, the presented data suggest manifestations of this phenomenon, which were conventionally categorized as cannibalism and cellulitis. The authors of the study reported a mortality rate of 24%, caused by lesions associated with these two conditions, ranking immediately after reproductive disorders (24.9%). Of the 542 carcasses examined, 56 deaths were explicitly attributed to cannibalism, and by extrapolation, approximately 74 cases of cellulitis can be estimated. The lesions leading to cellulitis were primarily located in the occipital region, but also on the flanks and thighs, being characterized by skin discontinuity, necrosis, and purulent lesions. In many cases, these lesions had already been subjected to ante-mortem cannibalism. The analysis of the distribution and typology of lesions in females suggests a direct link with mating behavior, indicating that they were, in fact, the result of sexually aggressive behavior exhibited by males. Even under conditions where measures to reduce trauma—such as claw trimming in males—were applied, lesions were observed on the trunk and thighs, in addition to those located on the dorsal region of the head. Therefore, these data provide clear evidence that forms of sexual aggressiveness were already present at that time, even if they were not explicitly conceptualized or named as such but were instead integrated into other pathological categories such as cannibalism and cellulitis [19].

The aim of this review is to explore the multifactorial etiology of male sexual aggression in poultry by synthesizing findings from ethological, physiological, and genetic studies. The hypothesis proposed is that this phenomenon is not the result of a single determining factor but rather the outcome of multiple interacting influences. Among these, intensive genetic selection for performance traits is considered a principal driver that alters normal mating behavior and contributes to the emergence of sexually aggressive patterns in males.

## 2. Methodology of the Review

This narrative review was developed using a structured literature search and selection strategy, designed to ensure methodological transparency and interpretative clarity. Relevant scientific publications were identified through comprehensive searches in four major databases: PubMed, Scopus, Web of Science, and Google Scholar. These platforms were chosen for their wide coverage of topics relevant to animal behavior, genetics, veterinary medicine, and poultry research.

The search strategy employed a combination of standardized indexing terms (such as MeSH in PubMed) and free-text keywords. Terms such as sexual aggression in poultry, genetic selection, mating behavior, courtship behavior, genetic aggressiveness, traumatic lesions, traumatic mating, and injuries in hens were used individually and in various combinations to optimize the retrieval of pertinent studies. The search process was iterative, with query refinements based on the volume and relevance of the results.

No publication date restrictions were applied. Articles were included based on their relevance to the topic, regardless of the year of publication. The final selection comprises both seminal studies that have shaped the field over time and more recent works that offer updated perspectives or contextual depth. Eligible sources included peer-reviewed original articles, review papers, and conference proceedings that focused on domestic poultry species, particularly *Gallus gallus domesticus*, or other avian species in which mating-related aggression has been documented. Studies were considered relevant if they addressed behavioral, genetic, physiological, or welfare-related outcomes associated with sexually motivated aggression. Only English-language publications with accessible full text were retained.

The screening process involved an initial assessment at the title and abstract level, followed by full-text evaluation of articles that met the inclusion criteria. Studies that were clearly outside the scope, such as those unrelated to aggressive or sexual behavior, or lacking scientific relevance, were excluded during the early phases of screening. Although formal dual screening was not conducted, the process was systematic, topic-focused, and reproducible.

**Figure 1:**
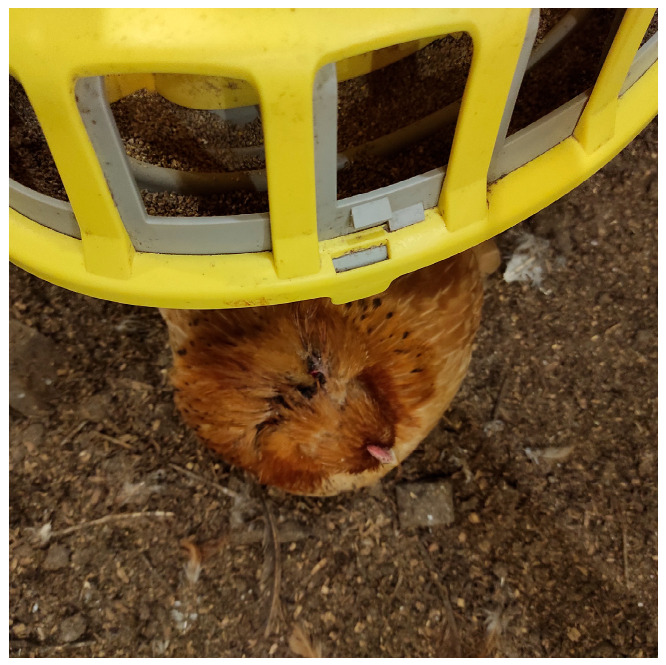
Hen exhibiting a traumatic lesion in the head region, concealed beneath a feeder. (Original photograph).

**Figure 2:**
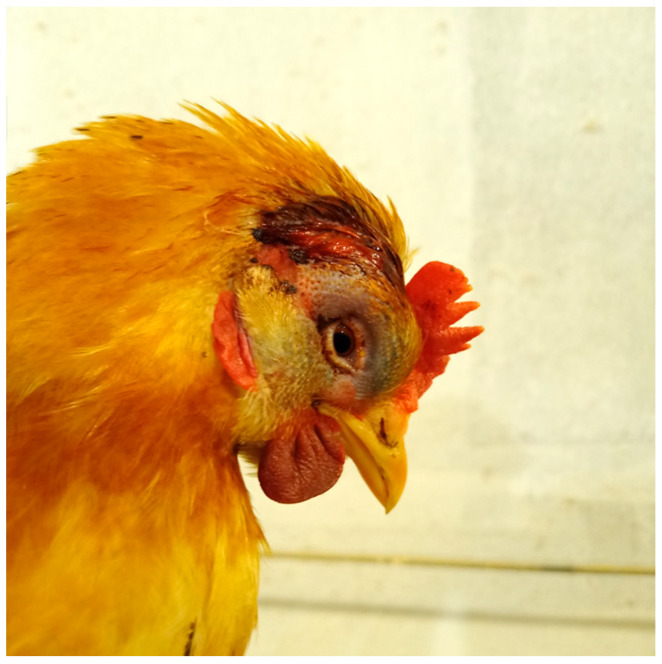
Hen, aged 23 weeks. The wound is located in the dorsal region of the head, slightly lateralized to the right side, immediately posterior to the comb. Marked swelling of the head, periorbital ecchymosis, and the presence of a serosanguinous exudate at the lesion site are observed. (Original photograph).

**Figure 3:**
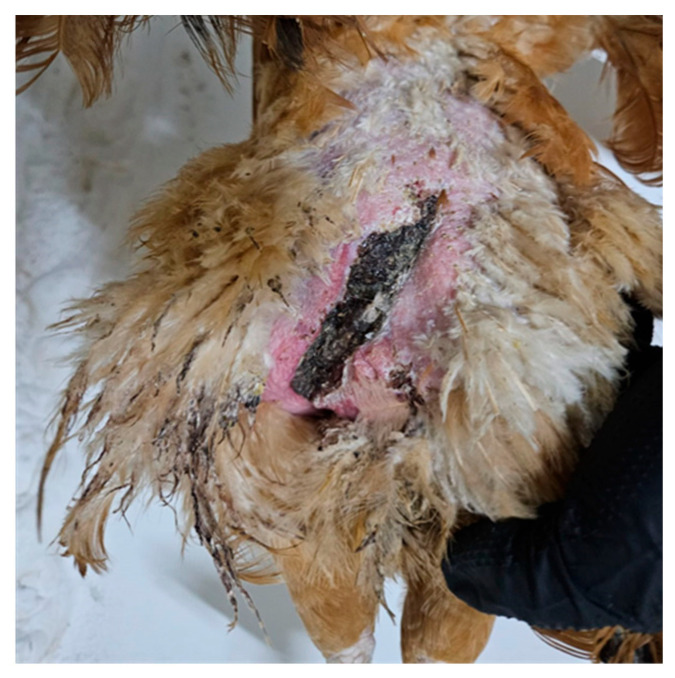
Hen, 40 weeks old. Traumatic lesion located on the left side of the body, extending from the dorsal trunk to the thigh. The incisional-like wound was caused by the shearing force of the rooster’s claws during a forced mating event. (Original photograph).

**Figure 4:**
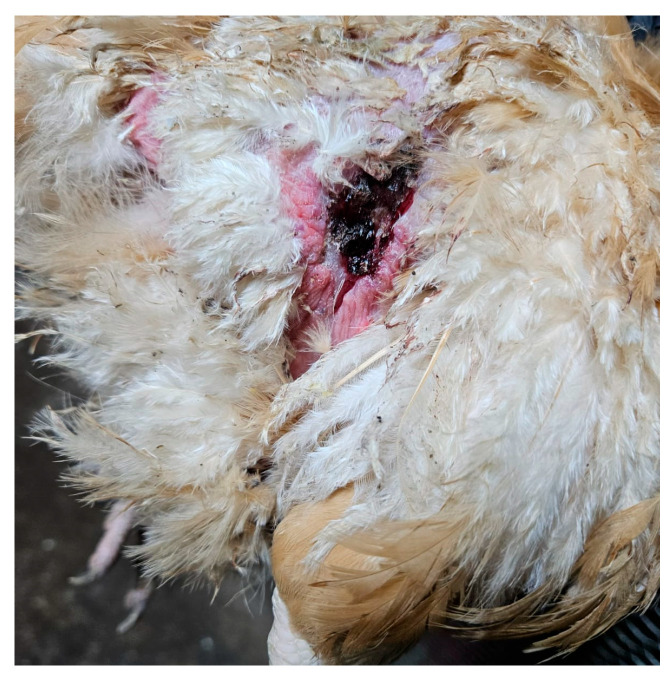
Hen, 40 weeks old, presents a traumatic lesion on the right side of the trunk, extending toward the thigh, in the process of healing. (Original photograph).

In addition to the published literature, the review is supported by original observational and experimental data collected by the authors in context-specific poultry populations. These contributions provide valuable insights and help supplement the published evidence. To illustrate the pathological outcomes of sexually motivated aggression, original photographic documentation of trauma-related lesions in hens is presented below (Figure 1, Figure 2, Figure 3 and Figure 4).

## 3. Characteristics of Reproductive Behavior in Roosters and Hens

Given the intrinsic connection between mating behavior and sexual aggression, key features of reproductive dynamics in domestic fowl are presented below to contextualize the emergence of aggressive patterns. To maintain optimal reproductive performance in broiler breeder hens, it is beneficial for sexual maturity to occur between 23 and 25 weeks of age [20].

A well-defined sexual dimorphism is present, characterized by more intensely colored plumage in roosters compared to hens, as well as greater physical development in males [21]. In a “natural” setting, where males and females are raised together, the early stages of sexual behavior begin as early as five weeks of age, while aggressive behavior emerges during the first week of life. It has been suggested that experience in fighting with other males, as well as interactions with females, influences the final organization of male reproductive behavior at maturity [2]. Certain neurobehavioral mechanisms associated with the mating process are present from the first days of life and are expressed even before the onset of sexual maturity [17].

**Figure 5:**
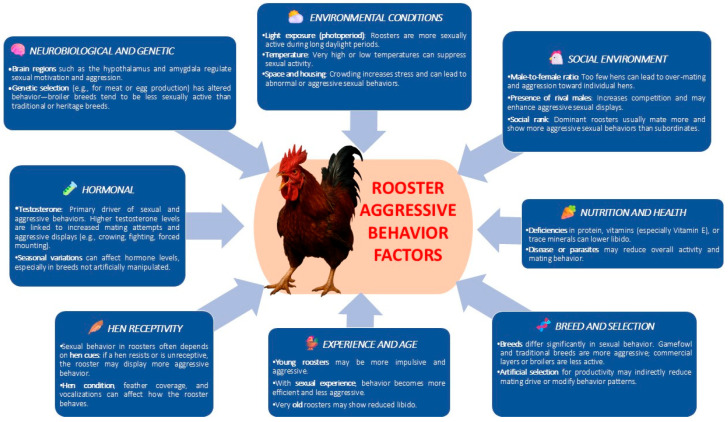
Factors influencing aggressive rooster behavior.

Reproductive traits in roosters are influenced by both management practices and inherent biological factors, with elements such as nutrition, housing conditions, and genetic makeup playing critical roles in determining fertility, libido, and mating behavior (Figure 5).

If males reach sexual maturity before females, the latter tend to exhibit avoidance behaviors, withdrawing in fear in response to mating attempts. Conversely, roosters that mature later may be subjected to female dominance, which can lead to inhibition of reproductive behavior. This form of “psychological castration” is considered irreversible [22].

Mating behavior follows a clear diurnal pattern, with a peak in the second half of the afternoon, when mating efficiency is also higher [23,24]. Mating frequency and efficiency differ substantially among roosters, but these behaviors do not consistently predict fertility outcomes. This observation points to the complex reproductive dynamics characteristic of large populations of breeding hens [17]. Semen quality, which can be influenced by factors such as age, frequency of collection, environmental conditions, and general management, also plays a critical role. These physiological variations may further modulate mating behavior and contribute to flock-level fertility outcomes [25,26].

In broiler breeder parent lines, the male-to-female ratio varies depending on the flock’s age. It typically starts at 10% when the males are 18 weeks old, decreases to 9% at 20 weeks, and further drops to 8% by 30 weeks of age [27]. Typically, a male-to-female ratio of approximately 1:10 or 1:11 ensures the highest fertility rates [17]. With the introduction of genetic lines characterized by high breast muscle yield, concerns have grown regarding male aggressiveness and its negative effects on mating behavior and fertility. Currently, a mating ratio of 1:12 is more commonly used than the previously predominant ratios of 1:8 or 1:10 [28]. However, in practice, this ratio tends to be closer to 1:20, as a significant number of roosters fail to fertilize hens [17].

An experimental study on Arbor Acres Plus broiler breeders in the post-forced molting phase (a procedure used to temporarily cease egg laying and stimulate reproductive system recovery) evaluated the influence of the mating ratio on reproductive and performance parameters. Three different ratios (1:10, 1:12, and 1:14) were compared in terms of egg production, fertility, hatchability, chick weight, and embryonic mortality. The results indicated that the 1:10 and 1:12 ratios had a significantly positive impact on fertility and total hatchability, without affecting other indicators such as laying rate, egg weight, or embryonic mortality. Therefore, the use of a 1:12 mating ratio is recommended to optimize reproductive performance and reduce the costs associated with maintaining males in broiler breeder flocks [29].

## 4. Mating Behavior and Reproductive Challenges in Modern Meat-Type Chicken Breeds

Compared to the mating behavior observed in Red Junglefowl (*Gallus gallus*) [2], it would have been expected, within the context of domestication, that the intensity of aggressive manifestations in the sexual behavior of domestic chickens would decrease significantly. However, research has not confirmed this trend [11]. Moreover, in certain situations, domestic males display incomplete and immature sexual behavior, often exhibiting aggression toward females who frequently do not respond positively to mating attempts [30]. In particular, it is unclear whether the absence of courtship behavior and excessive aggression are independent traits or genetically correlated. It is considered possible that these traits are genetically linked to morphologic characteristics of economic interest, such as the pronounced development of pectoral muscle mass, typical of lines selected by commercial poultry companies. Alternatively, these traits may be an indirect consequence of unsuccessful attempts to optimize fertility, especially toward the end of the annual reproductive cycle [17].

Although in natural environments aggressive behaviors of males toward females are rare [1,2,3], male aggressiveness may occur in specific contexts, such as immediately after a confrontation between two roosters or as a response to aggressive behavior shown by a hen toward a rooster [2]. In fact, Wood-Gush [1] documented that, over time, male aggression transferred to sexual behavior, suggesting an undesirable evolution of intersexual interactions in domestic settings.

Another factor influencing sexual behavior is the age of the birds. A comparative study conducted on two groups of birds with an age difference of four months revealed that this difference significantly impacts mating dynamics. Younger females exhibited greater sexual receptivity, and younger males performed twice as many matings as older ones. Predictably, fertility was higher in the younger flock [31].

Despite the physical dominance of males, the behavior of females plays a crucial role in the success of reproductive activity. This aspect is often overlooked in analyses of low fertility in meat-type chicken flocks [15]. As highlighted above, reproductive success in standard meat breeds remains at an acceptable level if sexual maturation is delayed until the age of 23–25 weeks. However, under stimulative photoperiod conditions, early maturation may result in smaller egg size at the beginning of the laying season, negatively affecting reproductive parameters. Furthermore, male precocity is often followed by a rapid decline in reproductive performance, necessitating partial or total replacement of males to maintain adequate fertility throughout the entire breeding season [20]. This strategy, known as spiking, involves the introduction of younger males into the flock to stimulate mating activity. However, this practice presents several challenges, including increased biosecurity risks, the potential introduction of pathogens, additional costs, and the destabilization of social hierarchies. It may also contribute to heightened stress and aggressiveness within the group [27]. As an alternative approach to support male reproductive performance in aging breeder flocks, nutritional strategies have gained attention. Supplementation with vitamins A and E has been associated with improved semen quality and delayed reproductive decline in roosters [32,33]. This may contribute to more consistent fertility over time and help limit the need for frequent flock interventions.

## 5. Courtship Behavior—A Fundamental Component of Reproductive Welfare in Chicken Populations

The courtship behavior of domestic birds, especially in hens, plays an essential role in reproductive success, although from the perspective of behavioral hierarchy, it is surpassed only by feeding behavior [34]. Duncan [17] proposes an analytical structure of sexual behavior divided into two distinct phases: appetitive and consummatory. Dysfunctions occurring in the appetitive phase, which include behaviors such as courtship, can affect the progression of the consummatory phase, which involves the actual act of mating. In studies on bird reproduction, the focus should not be placed exclusively on the moment of copulation, but also on the preceding sequences. Courtship, as part of the appetitive phase, has a direct impact on the likelihood of reproductive success, ensuring both stimulation and synchronization of the partners in order to align their cloacae precisely, which is essential for semen transfer in birds. Although instinctive, this behavior is complex and manifests through a ritual of specific gestures and postures, such as plumage display and courtship dance [21].

Unlike other species, the rooster does not develop a distinctive seasonal plumage during the reproductive period but may show a slight iridescence of the feathers, and the comb and wattles become firmer and more intensely colored [35]. Mate selection is not random but is influenced by both morphological traits and behaviors associated with courtship [17]. The courtship ritual includes actual or simulated food offerings, wing feather spreading directed toward the ground, circular movement around the female, and, if she is receptive, adoption of a characteristic posture followed by the act of mounting [17,21,35]. After copulation, the rooster returns to the ground, and the hen stands up and rearranges her feathers through a shaking motion. Although cloacal contact is not always visible, there are reliable indicators suggesting that it has occurred. Thus, the rooster abruptly ends the mounting act, making a noticeable backward and downward pelvic thrust, while the hen almost always displays a strong feather shake immediately after the rooster withdraws [17,21]. Reproductive performance varies by breed: males from light breeds may perform 30–50 matings per day, while heavy-breed males are limited to 5–10 [21].

However, the complete execution of courtship sequences requires adequate space and time; conditions which are often compromised in commercial operations with high stocking densities [11]. In such conditions, male behavior may be constrained, and females lack the space needed to avoid unwanted individuals. Courtship includes elements similar to those found in aggressive behaviors, which has led to the hypothesis that it may also serve an intimidating function [8].

In commercial systems, courtship behaviors are often absent, with mating occurring without preliminary stages [4,11,14]. Studies indicate a reduced presence of elements such as crowing or wing flapping, while other behaviors such as “waltzing” are almost completely absent [11]. Feather arrangement does not clearly guide behavior [8], wing flapping may signal aggression, and crowing may serve a location function within the group [11]. In a study conducted on a breeder flock belonging to slow-growing colored broiler parental lines, no cases of complete courtship behavior were observed throughout the monitored period. Courtship was absent in 51% of the mating attempts and incomplete in the remaining 49%. Interestingly, the lack of complete courtship did not appear to influence the evolution of hen receptivity over the course of the study [36].

There are significant differences among males in their attitude toward females: some adopt a protective behavior, guiding hens toward food or shelter, while others act aggressively and selfishly [37]. In certain cases, males show a preference for courting females of their own line, regardless of prior socialization experiences [37,38]. Additionally, the rearing system influences the frequency of sexual behaviors: in floor systems, courtship and female avoidance behavior are almost twice as frequent as in cage systems [39].

Comparative analyses of poultry genotypes show that sire-line broiler males perform a more restricted set of courtship acts and engage in forced matings more often than males selected for laying performance. These behavioral differences are attributed to the morphological constraints imposed by broiler growth traits, which limit their effectiveness during interactions with hens. Correspondingly, broiler breeder males in standardized trials tended to pursue hens directly instead of producing elaborate displays [5]. Millman and Duncan [5], building on earlier observations by Kruijt [2], argue that male courtship patterns are modifiable through learning and conditioning. Males from laying lines appeared to exploit this plasticity, refining their tactics between successive tests and thereby increasing their success in attracting females. Broiler males, by contrast, showed little comparable adjustment. This asymmetry in behavioral flexibility provides a plausible explanation for the hens’ eventual preference for laying-line males. Although the experiments uncovered no innate female bias toward either genotype, the authors contend that hens learned to avoid broiler males through repeated experience rather than instinct. Consequently, even though morphology and initial display behaviors seemed not to influence mate choice at first, repeated exposure to broiler males ultimately led to their avoidance.

## 6. Female Receptivity to Male Reproductive Behavior

The sexual behavior of the female in the context of avian reproduction is complex and multidimensional, being influenced by a range of behavioral, physiological, and environmental factors. According to Duncan [17], it is characterized by three essential traits: attractiveness, proceptivity, and receptivity. Attractiveness involves the female’s ability to attract the male’s attention, proceptivity refers to the initiation of sexual interaction by the female, and receptivity reflects her willingness to accept copulation.

Although hens usually respond positively to the rooster’s courtship behavior, under certain conditions they may adopt avoidance or rejection strategies. The male’s response to these behaviors varies depending on the social context and emotional state, manifesting either as tolerance or coercion. Although rare, roosters can also show preferences, choosing to mate more frequently with certain hens. Similarly, hens may actively select preferred partners [37]. Furthermore, there are hypotheses suggesting that females may selectively eliminate the sperm of non-dominant roosters, favoring alpha males [27].

Females may exhibit active attraction behaviors, positioning themselves in front of the male as a clear sign of copulation initiation. Sometimes this behavior occurs even in the absence of a male, being directed toward caretakers or other animals, suggesting an instinctive predisposition to trigger the mating sequence [35]. However, the role of the female in sexually aggressive behavior is not fully understood. Millman et al. [4] raised the question of whether male aggressiveness is a reaction to the absence of normal courtship behavior from the female, or a direct consequence of male aggression.

A study conducted in eight farms with the Ross 308 bird line monitored reproductive behavior between 20 and 28 weeks of age using direct observation and video recordings. It was found that some females do not recognize the intentions of males, and in some cases, refuse to adopt the squatting position, with mating taking place while standing. At the beginning of the laying period, receptivity rates are low, and this state persists even in later stages of the reproductive cycle. Male aggression is particularly observed after the initial mixing of flocks, and forcing hens to submit to copulation can lead to avoidance behavior and fear of males [11].

In some cases, courtship behaviors are not recognized by hens, which may explain the absence of sexual response or avoidance. This may be due either to low female receptivity or to ineffective courtship behavior. Research shows that hens display higher receptivity when housed with males from laying lines, responding positively to their advances, which translates into higher fertility rates. However, it is not clear whether this increased receptivity is due to the efficiency of courtship by laying line males or to an intrinsic preference for them [5].

The impact of male behavior on female choice has been documented in several studies [5,40]. Some hypotheses suggest that preference for a certain male may reflect a need for protection, not just a desire for reproduction. Studies have shown that females do not always recognize the health status of males, sometimes choosing sick males even after repeated interactions [5]. Leonard and Zanette [40] observed that, in the White Leghorn breed, there is no consistent correlation between mate choice and the size or color of the comb, although a tendency toward males with larger combs was noted.

Female avoidance behavior during mating attempts is considered a factor that may contribute to the emergence of male aggression toward them. It is possible that certain components of the courtship display are not perceived by females as clear signals, leading to an inadequate behavioral response. Additionally, reduced sexual receptivity in hens may at least partially explain their lack of cooperation during sexual interactions [5].

## 7. Growth Management Strategies and Their Impact on Aggressive Behavior

Light is a critical factor in regulating reproductive behavior and aggressiveness. Synchronizing sexual maturation between sexes, achieved through appropriate lighting programs, reduces the risk of aggressive male behavior [41]. If males are reared separately, synchronizing their photostimulation with that of the females becomes essential to ensure a similar onset of sexual maturity [28]. However, even under conditions of synchronized maturation, male aggressiveness may persist, being influenced by the higher mating frequency in intensive rearing systems compared to natural environments [41].

In addition to lighting, feeding has a significant impact on behavior. In a study assessing the impact of environmental factors during the rearing period of broiler breeder males, Custură et al. [42] found that variations in light intensity, bird density, and litter type did not significantly affect feed intake between 0 and 18 weeks of age. Although feed consumption remained stable across all conditions, such parameters remain relevant in designing optimal rearing environments for future reproductive performance.

Restrictive feeding regimes are necessary to prevent obesity in breeders but do not directly induce aggressive behavior. On the contrary, males that are overfed may exhibit more aggression than those subjected to restriction [17]. The use of low-nutrient-density feed and supplementation with tryptophan—a serotonin precursor—has shown calming effects [43], although their commercial application is limited due to costs [28].

A recent study evaluated the impact of a combination of qualitative feed restriction strategies (diluting the diet with 20% oat hulls and daily administration of fibrous feed) on the behavior and welfare of Hubbard M77 breeder males during the rearing period. Compared to conventional restriction, the tested treatment did not result in significant improvements in welfare indicators such as behavioral responses to frustration, tonic immobility, or the presence of stress bars on feathers. Although birds in the experimental group showed slightly fewer frustration-related behaviors, these effects were not consistent over time. The results support the conclusion that qualitative feed restriction does not provide clear and reproducible benefits in improving the welfare of broiler breeder males [44].

In recent decades, the essential role of a balanced gut microbiota in maintaining overall health and behavioral stability in poultry has become increasingly evident, prompting a growing body of research into how microbial composition may influence aggression-related outcomes. Studies in both broilers and laying hens have shown that manipulating the gut microbiota can reduce aggressive behaviors [45,46,47]. While these findings are primarily based on models of general or stress-induced aggression, they underscore the behavioral relevance of gut microbial balance. Nutritional interventions such as phytogenic additives, protein-source reformulation, and probiotic supplementation, commonly used as part of growth management strategies, have also been shown to support microbiota stability and enhance physiological resilience in poultry [48,49,50]. In breeder flocks, where aggression often emerges in the context of mating, these microbiota-targeted approaches may hold potential for influencing sexually motivated aggression as well. Although direct evidence is lacking, it is reasonable to consider that growth strategies affecting microbial composition could, indirectly, contribute to modulating behavioral responses during the reproductive phase. Rearing technology, including sex separation, stocking density and the age at which males are introduced into the female flock, strongly influences the expression of sexual and agonistic behavior. Studies have shown that birds housed together during the rearing period displayed lower aggressiveness during mating compared to those reared separately [2,11]. Agonistic activity among females was 70% higher in flocks without males than in those with males. Females reared in cages under crowded conditions exhibited 30–50% fewer agonistic acts than floor-reared hens in lower-density flocks. In males, both genetic lines and rearing environment affected the frequency of aggressive behavior. Males reared on the floor exhibited nearly four times more aggressive acts than those reared in cages [39].

Another management factor influencing behavior is the procedure of beak, claw, and spur trimming in males, performed to prevent injury to females during copulation. Although controversial from an animal welfare perspective, this practice is commonly applied to breeding males and should be performed during the first days of life [12,41,51,52,53].

## 8. Sexual Aggression and the Role of Selection in Optimizing Productive Performance

Intensive genetic selection aimed at maximizing productivity in breeder hens has generated numerous side effects on behavior, including an increase in male sexual aggressiveness. These violent manifestations during mating directly affect the health and welfare of females, leading to injuries, chronic stress, and reduced reproductive performance. Thus, integrating behavioral traits into selection programs becomes essential for balancing production goals with animal welfare objectives [16,54]. Behavior, physiology, and morphology are closely interconnected, forming a complex system of traits that determine the adaptability of birds to different production environments. The homeostatic characteristics of each genetic line can be analyzed to understand the influence of genetic factors on physiological functions regulated by biogenic amines, with direct impact on productivity and longevity. Although selection focused on production traits has negatively affected mating behaviors, recent research indicates the possibility of balanced selection that supports both productivity and welfare [54].

Examples from other species, such as turkeys—where natural mating has been replaced by artificial insemination due to morphological changes induced by selection—highlight the limits of unilateral selection. In laying hens and meat-type birds, natural mating is still predominant, but difficulties in maintaining fertility in meat breeds are increasing, reflecting the imbalance between selection for production traits and the neglect of reproductive traits [16]. For example, selection for broiler production initially focused on body weight gain and feed conversion efficiency. In recent years, selection criteria have been expanded to include factors such as survival rate, body conformation, skeletal integrity, and other relevant traits [55].

A comparative study between breeds genetically selected for meat and egg production revealed a significantly higher incidence of non-reciprocal aggressive behavior in meat-type birds. These birds exhibited higher serum concentrations of testosterone and estradiol, as well as greater density of androgen receptors in the hypothalamus. The results suggest that genetic selection for rapid growth is associated with hormonal and neurobiological expression patterns that promote increased aggressiveness [18]. Such alterations support the hypothesis that selective breeding can influence hormonal expression and, consequently, disrupt the normal development of reproductive behavior, which is evidently hormonally regulated and modulated by photoperiodic cues. However, selection-induced changes in endocrine pathways may interfere with this natural regulation, leading to maladaptive behavioral outcomes.

In meat-type parent lines, maintaining fertility and achieving satisfactory hatchability rates is becoming increasingly problematic. These difficulties can be directly attributed to selective breeding focused on production traits in the offspring, combined with a lack of selection for traits relevant to mating behavior. The reproductive issues observed in males from broiler breeder lines are most likely the consequence of unilateral selection strategies centered on performance. Intensive selection for growth-related characteristics has altered the courtship behavior of roosters, rendering it incomplete or entirely absent [16]. The reduction in these courtship behaviors appears to be directly associated with the emergence of sexual aggressiveness, as males bypass the normal approach sequences toward females, thereby increasing the likelihood of forced copulations and physical injury to the hens.

Duncan proposed the hypothesis that certain behavioral traits such as sexual aggressiveness or poor mating performance may be genetically correlated with production-related traits, particularly those heavily selected in commercial breeding programs such as broad-breasted conformation. Furthermore, he suggests that the increased sexual aggression observed in broiler breeder males may be the unintended outcome of a misguided selection strategy aimed at enhancing fertility. Specifically, the decline in fertility observed toward the end of the reproductive cycle was erroneously attributed to reduced libido. Consequently, males exhibiting rapid and direct approaches toward females may have been preferentially selected under the assumption that such behavior indicated heightened sexual drive. However, in reality, this behavioral pattern may reflect elevated sexual aggressiveness. This hypothesis is further supported by the observation that broiler males frequently lack key courtship behaviors such as titbitting, a display in which the male temporarily withdraws from the female to encourage voluntary participation in mating [17].

## 9. Genetic Foundations of Male Aggressive Behavior Toward Females

Although a range of environmental factors—such as stocking density, social hierarchy, lighting type, restricted feeding, or group composition (single-sex or mixed-sex)—can influence levels of aggressiveness, increasing evidence suggests that the predisposition to aggressive behavior has a strong hereditary component (Figure 5) [24].

In poultry farming, breeding programs are evolving from progeny testing to genomic selection combined with indirect genetic effects, thereby significantly reducing the generation interval [54]. A genetic basis for male-to-female aggression has been previously suspected, given that breeder males from broiler parent lines exhibit less pronounced courtship behavior compared to males from laying lines or fighting breeds [7]. Although agonistic behavior between pairs of males is significantly influenced by genetic line, no genetic line effects have been detected in the frequency of agonistic acts among females [39]. Aggressive behavior and sexual drive in roosters were investigated by Siegel [56], who provided strong evidence of the influence of additive genetic control over avian aggressiveness.

In this context, a recent study published by Li et al. [24] marks a major advancement, representing the first genome-wide association study (GWAS) dedicated to aggressiveness in roosters. Using a high-density genotyping platform (600K SNPs), the authors explored the relationship between genetic variation and aggressive behaviors in 265 males from a local “yellow dwarf” breed. Aggressive behavior was quantified using standardized behavioral parameters (number of fights, frequency and duration of aggressive acts), alongside assessments of growth performance and feed intake. The results identified 33 SNPs significantly associated with male aggressiveness, with one marker located in an intron of the *SORCS2* gene (rs312463697) showing a strong and consistent association with aggressive behaviors, including those of a sexual nature. This gene, known for its role in the development and function of the central nervous system, encodes a membrane receptor involved in neurotrophin signaling and the regulation of dopaminergic circuits—crucial for the control of impulsivity, motivation, and social interactions.

Furthermore, gene expression analysis in pituitary tissue revealed that males exhibiting the most aggressive behaviors had significantly higher levels of *SORCS2* expression compared to the most docile individuals. These findings support the notion that excessive activation of this gene in key brain regions may lead to the hyperstimulation of neural networks responsible for sexually aggressive behavior, which aligns with observed field manifestations. Therefore, this study provides compelling evidence that sexual aggressiveness in roosters has a determinable genetic basis, with *SORCS2* emerging as a key candidate gene in the regulation of this trait. The identification and understanding of these molecular mechanisms open new avenues for assisted genetic selection, offering the potential to reduce undesirable aggressiveness in commercial populations and sustainably improve both animal welfare and the efficiency of poultry breeding systems.

A recent study using transcriptomic and metabolomic analyses identified several molecular mechanisms associated with aggressive behavior in Henan gamecock males. The results indicated overexpression of genes involved in serotonergic and dopaminergic pathways, correlated with negative regulation of genes related to neuro-immune function. Additionally, several previously unknown genes with brain-specific expression were identified, which may play a key role in the manifestation of aggressiveness. These findings support the hypothesis that avian aggressiveness is underpinned by a complex reconfiguration of neurochemical and immune signaling networks in the brain [57].

## 10. Description of Lesions Specific to Sexual Aggression

Regarding the description of lesions caused by sexual aggression, the specialized literature provides few details, with more emphasis placed on behavior and its causes than on anatomopathological aspects. Normally, hens benefit from the protection offered by a dense layer of feathers, which not only plays a thermoregulatory role but also shields them from potential injuries caused by the rooster’s claws during mating [41]. Lesions are frequently located on the head, trunk, and under the wings, where the skin is traumatized by the claws and beak of the males during forced mating behavior. The same hen may present with multiple lesion sites [14]. Head lesions were recorded in a small proportion of affected hens—3.26% of the flock—with injuries primarily located on the back of the head, followed by the comb and earlobes. Notably, such lesions were observed exclusively during the first five weeks after the onset of laying. Injuries on the comb and earlobes were typically seen during the early stages of the phenomenon. The dimension of these lesions varied, with most measuring between 0.5 and 1 cm, while larger wounds exceeding 1 cm were observed less frequently [36].

Based on the morphological changes observed in affected tissues, lesions resulting from mating extend sequentially from the integument through the subcutaneous connective tissue and into the superficial muscle layers. Once the skin is torn, its edges retract—a vital local response attributable to contractile muscle fibers embedded in the dermis. As part of the host’s vital response, a peripheral rim of fibrin is deposited around the wound, demarcating the lesion from adjacent healthy structures [58].

The gross morphology of trauma associated with sexually aggressive behavior varies considerably depending on both the intensity of force applied and the frequency of repeated episodes. Lesions range from superficial wounds with smooth, regular margins to deep lacerations or avulsions marked by irregular, frayed edges and exposed subcutaneous or muscular layers. These features are commonly accompanied by areas of dry necrosis, particularly at the wound edges and exposed dermal surfaces, as well as by superficial abrasions of the underlying musculature. In many cases, the wound surface is contaminated with litter debris, suggesting chronicity and inadequate mechanical protection. Post-mortem examinations routinely confirm these lesions, which reflect a progression from initial integumentary disruption to widespread inflammatory involvement and, in severe cases, can extend over large areas of the thigh, potentially impairing locomotion [14,36].

Although the study on mortality causes in breeder hens does not explicitly refer to sexual aggression, the topographical distribution of the lesions closely matches those typically associated with this behavior. Moreover, the authors mention that the injuries are related to mating activity. This adds significant value to the morphological characterization of the lesions: the injuries predisposed to cellulitis were defined by cutaneous disruption, tissue necrosis, and the presence of purulent exudate. In many cases, these lesions had already been subjected to ante-mortem cannibalistic pecking [19]. These findings provide important insights into the pathogenesis of trauma-related infections in poultry and underscore the potential impact of sexually aggressive behavior on the welfare and health of breeder flocks, as in severe cases, such behavior may even culminate in the death of affected females [14,17]. These traumatic lesions can be exacerbated by deformities in the toes of roosters, by improperly trimmed claws, or by sexing errors [22].

A key aspect in the assessment of traumatic lesions in domestic birds is the clear distinction between injuries resulting from sexually aggressive behavior and those caused by cannibalism. In the context of sexual aggression, lesions are characterized by tearing of the skin and muscle tissue, though without significant tissue loss—an element that defines lesions caused by cannibalism [14,59].

Another important differentiating criterion is the mechanism of trauma production. In cases of sexual aggression, lesions occur during forced copulation, when the male causes skin injuries using his claws and beak [14]. By contrast, cannibalism is preceded by aggressive pecking behavior, whose etiology is multifactorial, involving environmental, nutritional, and behavioral factors [59,60,61]. Cannibalism can also be a learned behavior and is enhanced by the flock observing other birds engaging in same behavior [59].

The only common element between these two forms of deviant behavior is their classification within the category of ethological disorders, both reflecting a dysfunction in the species’ natural behavioral pattern. In some cases, injuries caused by sexual aggression may become targets of ante-mortem cannibalism. These behaviors are not mutually exclusive and may occur simultaneously [19].

## 11. Environmental Stressors and Their Role in Triggering Sexual Aggression in Poultry

Sexually aggressive behavior in poultry is a multifactorial phenomenon, shaped by the interaction between genetic predisposition and environmental or nutritional factors. Stressful conditions commonly encountered in intensive farming systems can promote the expression of sexual aggression, which in turn contributes to a general decline in flock welfare. Although most of the stress-related factors discussed below have been addressed individually in previous sections, this chapter aims to consolidate and interpret them through the specific lens of sexual aggression, highlighting their synergistic role in triggering or exacerbating this behavior in intensive poultry systems.

As discussed in the genetics section, sexual aggression has a clear hereditary component. The *SORCS2* gene is one of the strongest candidates associated with aggressive behavior, influencing dopaminergic signaling pathways [24]. However, genetic predisposition alone is not sufficient; it can be amplified by a series of environmental stressors.

One major factor is photostimulation. When applied excessively or incorrectly, it can lead to a loss of synchronization in sexual development between males and females. Males that reach sexual maturity too early may attempt forced mating with physiologically unprepared females, resulting in trauma and coerced behaviors. Conversely, when females develop earlier than males, psychological castration of the males may occur, leading to behavioral inhibition [27,41].

High stocking density is another environmental stressor with significant behavioral impact. In overcrowded spaces, males are unable to perform normal courtship rituals, which may lead to miscommunication with females [11]. This often results in chasing and forced mating. Furthermore, an excessive male-to-female ratio promotes overmating, causing feather damage, skin lesions, and considerable stress within the flock [27]. Additionally, in high-competition settings, males may perceive courtship as a disadvantage, since it increases the likelihood of losing access to the female to a rival, and may skip it altogether, further exacerbating aggression.

The practice of sex-separate rearing can also contribute to sexual aggression, particularly at the point of flock merging. Males raised in isolation tend to display more intense aggressive behavior, especially during the early stages of cohabitation with females [2,11].

Nutritionally, unrestricted feeding has been linked to more aggressive behavior in males compared to those under controlled-feeding regimes [17]. Nevertheless, supplementation with the amino acid tryptophan has shown beneficial effects in reducing aggression, despite its higher cost [28,43]. Additionally, the emerging role of gut microbiota in behavioral regulation suggests a possible indirect influence on sexual aggression, particularly in genetically predisposed or environmentally stressed birds [45,46,47].

Certain preventive interventions can reduce the injuries caused by sexually aggressive behavior. These include spur trimming, beak cauterization, and partial toe removal—procedures that lessen trauma severity, although they may themselves cause stress if improperly applied [12,41,51,52,53].

Another important factor is spiking, the introduction of young males into existing flocks to boost fertility. Although reproductively effective, this practice causes significant social stress, disrupts established hierarchies, and, when too many young roosters are added, can lead to severe episodes of overmating [27].

All these elements underscore the fact that environmental stress, even when not the primary cause of sexual aggression, acts as an amplifier, especially in individuals with genetic susceptibility. The interplay between environmental stressors, nutritional imbalances, and genetic predisposition appears to shape the expression of sexually aggressive behavior in poultry. While genes such as *SORCS2* have been linked to aggression-related traits, external factors including high stocking density, improper photostimulation, and trace element deficiencies may act as amplifiers of these behaviors. Understanding these multifactorial correlations is essential for both veterinary medicine and breeding strategies as it may guide targeted interventions aimed at reducing trauma-related injuries and improving overall flock welfare and productivity.

## 12. Conclusions and Future Research Direction

Sexual aggression in roosters from broiler breeder lines, including both conventional and slow-growing genotypes, is a direct consequence of intensive genetic selection focused primarily on productive traits.

This behavioral imbalance typically becomes evident around the onset of sexual maturity and is shaped by social dynamics, asynchronous sexual development between sexes, and environmental conditions.

Morphological changes associated with growth-oriented selection, combined with the suppression or loss of natural courtship behavior, have led to the emergence of aggressive mating patterns that deviate from ethological norms.

The absence of adequate courtship disrupts sexual synchronization and increases the likelihood of coercive mating, which in turn triggers stress, fear, and avoidance behaviors in hens. Reduced female receptivity perpetuates conflict, undermines fertility, and compromises overall reproductive success. Environmental factors such as lighting programs, stocking density, feeding regimes, and gut microbiota composition also modulate the expression of sexually aggressive behaviors, highlighting the potential of targeted management interventions.

The intensification of these behaviors through unilateral genetic selection underscores the need to incorporate behavioral traits into breeding objectives. Recent genomic studies, including genome-wide association analyses, confirm a strong hereditary basis for sexual aggression. Candidate genes such as *SORCS2*, involved in neurobiological pathways linked to impulsivity and social interaction, offer promising targets for assisted selection aimed at reducing undesired aggression in breeder males.

The pathological consequences of this aggression are evident through specific traumatic lesions, typically on the head, trunk, and thighs, that impair the welfare, productivity, and longevity of hens. These injuries illustrate the severity of the problem and the necessity of standardized lesion monitoring and behavioral assessments.

Future research on sexual aggression in broiler breeder lines should focus on combining genomic selection with a better understanding of environmental influences. Although recent studies have identified genetic markers associated with aggressive behavior, such as *SORCS2*, their consistent phenotypic expression may depend on the rearing environment. Factors such as stocking density, lighting conditions, nutrition, and social dynamics are known to influence behavioral outcomes and should be systematically considered when applying genomic selection in practice.

At the same time, multifactorial models that integrate genetic data with behavioral observations and environmental variables could improve selection accuracy. Such models would support the development of breeding strategies that not only reduce the genetic predisposition to aggression but also enhance birds’ behavioral stability under common production challenges.

Further work should also investigate how management interventions, such as enrichment, lighting schedules, or dietary adjustments, can interact with genetic background to reduce the expression of sexually aggressive behavior. A clearer understanding of these interactions will help optimize both selection and housing strategies.

In addition, specific attention should be directed toward the genetic basis of courtship behavior. The near absence of courtship rituals in modern broiler breeder males raises the question of whether this behavior has been unintentionally reduced or eliminated through past selection for production traits. Genome-wide association studies (GWAS) targeting courtship-related behaviors could help determine whether these behaviors are genetically linked, positively or negatively, to production traits such as growth rate, muscle mass, or feed efficiency. If such traits are negatively correlated, it is possible that courtship behavior has been unintentionally suppressed through past selection focused solely on productivity.

Another important direction would be the investigation of female receptivity and its potential genetic or physiological determinants. While current research has primarily focused on male behavior, reduced female cooperation during mating may also contribute to sexual conflict and decreased fertility. Future studies could explore whether individual differences in receptivity are associated with hormonal profiles, stress response, or subtle genetic variation, and how these factors interact with male behavior and environmental conditions.

In summary, future efforts should move toward integrative selection programs that address both inherited predispositions and modifiable environmental triggers of aggression. Expanding genetic studies to include courtship and female behavioral responses will provide a more complete foundation for optimizing welfare, fertility, and long-term sustainability in broiler breeder production systems.
